# Blind alleys and dead ends: researching innovation in late 20th century surgery

**DOI:** 10.1136/medhum-2016-011176

**Published:** 2018-01-05

**Authors:** Harriet Palfreyman, Roger L Kneebone

**Affiliations:** 1 Centre for the History of Science, Technology and Medicine, University of Manchester, Manchester, UK; 2 Imperial College Centre for Engagement and Simulation Science, Imperial College London, London, UK

**Keywords:** history, surgery, performance

## Abstract

This article examines the fortunes of one particular surgical innovation in the treatment of gallstones in the late 20th century; the percutaneous cholecystolithotomy (PCCL). This was an experimental procedure which was trialled and developed in the early days of minimally invasive surgery and one which fairly rapidly fell out of favour. Using diverse research methods from textual analysis to oral history to re-enactment, the authors explore the rise and fall of the PCCL demonstrating that such apparent failures are as crucial a part of innovation histories as the triumphs and have much light to shed on the development of surgery more generally.

In February 1988, *The BMJ* published an article on percutaneous cholecystolithotomy (PCCL), a new surgical procedure for removing gallstones.^1^ Eight patients had undergone the PCCL, which involved extracting the gallstones using an endoscope introduced through a small puncture in the abdomen; a much less invasive alternative to the standard open operation of cholecystectomy, in which the whole gallbladder was removed, stones and all. Although highly innovative at the time, the PCCL is all but unknown today. The 1988 *BMJ* paper came at a time when surgery was undergoing revolutionary change due to the emergence of ‘minimally invasive’ or ‘keyhole’ procedures. Rather than making large incisions, minimally invasive techniques instead required a few small punctures into the body through which the surgeon operated using endoscopic instruments. Despite initial controversy, minimally invasive surgery quickly became widespread with procedures generating enormous excitement among surgeons and patients.^2^ The authors of the 1988 paper on the PCCL—radiologist Michael Kellett and surgeons John Wickham and Christopher Russell—concluded their piece by suggesting the minimally invasive PCCL would be ‘a vital adjunct’ to other treatments for gallstones.^1^ In the years following this article, however, only a handful of further publications on the procedure appeared.^3–5^ Instead, attention quickly turned to a different procedure for treating gallstones; laparoscopic cholecystectomy. By removing the entire gallbladder laparoscopically, this procedure represented a ‘significant milestone’ in the development of minimally invasive surgery and left the PCCL languishing at a dead end.^6^

The history of minimally invasive surgery has been largely written by its practitioners.^7–9^ As such, it is rich in biographical detail and accounts of its most successful operations; however, there has also been a tendency to focus only on the triumphs. In contrast, historians of medical innovation have long called for studies that take a more nuanced view of such narratives.^10 11^ This article addresses that call and shows that the emergence of minimally invasive surgery was a complex process marked by the successes of operations like the laparoscopic cholecystectomy and by short-lived, experimental procedures that turned out to be much less transformative than initially hoped. With this aim, we explore in more depth the PCCL, one of the blind alleys down which clinicians went within a climate of experimentation. We examine why and how the PCCL emerged; what problems it addressed; how it developed within a landscape of other treatments and finally, why it eventually fell out of favour. In order to do this, we required a source base beyond the medical journal articles, we began with here. From such papers we can access descriptions of the technique and reports of patient outcomes, but it is difficult to discern why and how the PCCL developed in the first place. In particular, it is difficult to recapture the social, political and professional context of the times, since this is seldom, if ever, described in scientific reports. To address the limitations of published sources, we have turned to research methods that draw on approaches from both of our respective fields. One of us (HP) is an historian of medicine while the other (RLK) is a surgeon and surgical educator who trained as a general surgical registrar in the 1980s. In researching the PCCL, we have conducted an extensive series of individual and group oral history interviews with key figures who worked on developing the PCCL.

In addition, we have sought to reveal elements of the PCCL story that interviews alone cannot. In order to document the tacit, embodied practices which underpin surgical procedures we have used simulation-based re-enactment (SBRE), a methodology developed by Roger Kneebone and Abigail Woods, to augment the more orthodox historical methods described above.^12^ By restaging a now lost procedure we can better capture the practice of the operation, the performance styles of the operators and the different roles played by each team member than by using textual sources or oral accounts. Furthermore, this physical and experiential practice serves to trigger memories for the participants that may not have arisen in the oral history interviews. Our re-enactments brought together clinicians Kellett, Wickham, Russell and others to ‘re-enact’ a PCCL using physical simulation. Simulations were followed by group interviews, acting as a prompt for further recollections and detail. In the following article, we weave together discussion of the history and our research methodologies, exploring the blind alleys and dead ends often overlooked in studies of medical innovation.

## Emerging interests: oral history and the origins of the PCCL

Oral histories have long been used by medical historians to access the narratives and perspectives of people (such as nurses and patients), whose experiences are not adequately represented in official written records.^13^ However, oral history interviews can also be a valuable tool in researching the roles of those who *do* appear in these orthodox sources. In her work on the history of health policy, historian Virginia Berridge argues that, despite going against a typical ‘history from below’ approach, interviewing those in powerful positions can add much to our understanding of an event or time.^14^ Indeed, although most of the interviewees in our study had numerous publications to their names, the conventions of such texts offered a limited narrative of the emergence of minimally invasive surgery. Medical journals and textbooks can tell us how an operation was performed or what its success rate was, but they tell us little about how the operation was initially developed. In order to explore these elements, the oral history interview is a crucial research tool. Between 2012 and 2014, we interviewed numerous clinicians who were at the forefront of developing minimally invasive techniques in the UK.^2^ Out of these interviews emerged the story of the PCCL.

One of our interviewees was retired urological surgeon John Wickham who worked extensively on minimally invasive techniques during his career. From his early surgical training, Wickham had become increasingly concerned with the iatrogenic damage inflicted on patients by large operative incisions. Early on it was nephrolithotomy (open surgery to remove kidney stones) that drew his particular ire. Wickham described the standard procedure for removing large kidney stones in the 1970s as particularly invasive. "You took a knife, slit the kidney down, opened it—as for grilling—picked the stone out, stitched it up like the weekend sirloin, let go and hoped it didn’t bleed too much—which it usually did!" (Wickham, personal interview, 21 June 2013) The lengthy cut needed to access the kidney and the large incision into the organ itself often resulted in long postoperative recovery times accompanied by a loss of kidney function. Eager to reduce the problems caused by such large incisions during the nephrolithotomy, Wickham began working closely with Michael Kellett, a radiologist colleague at the Institute of Urology in London. In the late 1970s, Kellett had started performing a new procedure to drain obstructed kidneys using a tube inserted through the patient’s loin. Shortly after, Kellett and Wickham heard about an experimental procedure in Germany where surgeons had removed a kidney stone through the narrow tract created by such a procedure.^15 16^

This German procedure (percutaneous nephrolithotomy (PCNL)) was a considerable undertaking, beginning with the radiologist inserting a needle, guided by X-ray, into the kidney. A guidewire then took the place of the needle and over this wire a series of dilators, increasing in size, enlarged the diameter of the tract in a multistage procedure taking several weeks. Once the tract was large enough, the surgeon could remove the stone using an endoscopic surgical (Dormia) basket. Kellett and Wickham developed a single-stage version of this procedure and performed their first one in November 1979, which met with great enthusiasm. As Wickham recalled, "I whipped a cystoscope in with a Dormia basket, grabbed the stone, yanked it out, then the theatre all burst into clapping! So I thought, well, this is it, we must develop endoscopic renal surgery, which is where we went from". (Wickham, personal interview, 21 June 2013) Wickham and Kellet published their first article on this procedure in the *BMJ* in 1981 recording their conviction that the PCNL ‘will rapidly become established as the expected norm for the removal of most renal calculi and that the considerably more traumatic access operation presently used will become obsolete'.^17^ Indeed, the PCNL was widely adopted and soon became standard practice for removing kidney stones in suitable cases. This was a time of rapid change within stone surgery, and in due course PCNL was followed by the development of extracorporeal shock wave lithotripsy (ESWL), an even less invasive approach which fragmented kidney stones using targeted shockwaves from outside the patient’s body. Stone fragments were then passed in the urine without the need for any further intervention. By the late 1980s, PCNL and ESWL were common procedures.^2^

Having met with success in the kidneys, Wickham began considering the possible applications of minimally invasive surgery in other areas of the body, wondering in particular about gallstones. The standard treatment for gallstones at the time was open cholecystectomy—the removal of the gallbladder—which, to Wickham, represented another unnecessarily damaging operation. Kellett explained that in some instances patients did not respond well to gallbladder removal and suffered unpleasant residual symptoms. "There is a post-surgical syndrome; you get discomfort, indigestion, and (Wickham) thought ’well the gallbladder’s there for a purpose, the stone isn’t. If you get the stone out and leave the gallbladder there it might be okay'". (Kellett, personal interview, 8 December 2014) Indeed, one 1987 article in the journal *Gut* reported that after evaluating 93 cholecystectomy patients 2 years following their surgeries that 44 of them were suffering from ‘postcholecystectomy symptoms’, including flatulence, abdominal pain and diarrhoea.^18^ Until the 1980s, open cholecystectomy had been considered the gold standard in the treatment of gallstones.^19^ However with the development of non-surgical treatments for kidney stones, such as ESWL, this was a period of new uncertainty about how best to treat gallstones. There were hopes that ESWL or drug therapies to dissolve stones in the body with no need for any surgical intervention would prove the future of gallstone management.^20^ For Wickham and Kellett, it was the percutaneous procedure they had developed for the kidneys that could perhaps challenge the open cholecystectomy and so they began working on ways in which they could apply the PCNL procedure to the gallbladder.

Initially, they encountered some difficulty on account of anatomical differences between the kidney and the gallbladder. Kellett explained that ‘(the) trouble there was (that) the gallbladder’s very mobile, it’s very floppy, it moved away all the time so putting dilators in was rather difficult at first. When you got used to it, no problem'. (Kellett, personal interview, 8 December 2014) These characteristics of the gallbladder eventually required the practised hand of a skilled radiologist and some specialist mechanical intervention. Here, the clinicians turned to their links with industry, beginning a collaboration with Stuart Greengrass who worked for Olympus KeyMed, a leading manufacturer of endoscopic equipment in the UK. Greengrass explained that Wickham first approached him with some ideas for instrumentation that would negate the problem of the ‘floppy’ gallbladder.

To do that (Wickham) said “well what we could do is we go in through with a little laparoscope here, we can grab hold of the gallbladder, we can pull it to the abdominal wall, we can make an incision in it and we can pull the stones out and then stitch it up and then let it go back where it should be". And I thought cor, this is interesting, I wonder if we can do something with this. (Greengrass, personal interview, 3 December 2014)

During this development process, Greengrass would often visit the hospital, observing operations and working closely with the clinicians on the design of such new, specialist instruments. This relationship with industry was far from the only collaboration this new procedure would demand.

Although Wickham had undergone general surgical training, as a urological surgeon, the gallbladder fell outside his specialism so he began working with Christopher Russell, an upper gastrointestinal surgeon at the Middlesex Hospital. In the early, uncertain days of minimally invasive surgery though, this collaboration—a urologist working with a hepatobiliary surgeon—drew criticism. From the first half of the 20th century, Britain had seen a significant increase of medical and surgical specialisations.^21^ Whether focused on a specific anatomical territory (ie, orthopaedics, neurosurgery, urology) or on a specific patient groups (ie, paediatrics, gerontology) new specialties and subspecialties had, by the late 20th century, come to dominate in Britain’s hospitals.^22^ Practitioners were keen to claim legitimacy and prestige for their own new specialties and could be protective over what they saw as their turf. While anatomically the gallbladder fell under the remit of the relatively new subspecialties of upper gastrointestinal and hepatobiliary surgery, it is easy to see how a urologist would become interested in the organ. Urology was a specialism that had its historical roots in stone surgery; the long history of treating bladder stones through lithotomy was one operation on which urology staked its claim as a specialty. Indeed, the Institute of Urology, at which Wickham and Kellett worked, was based partly at St Peter’s Hospital in London, which had been founded in 1860 as a specialist hospital devoted to treating bladder stones.^23^ Several specialisms then could feasibly claim the gallstones as an object of their expertise, a situation which naturally could prove contentious. This competition between specialisms, coupled with the fact that Wickham and Russell were advocating for new, minimally invasive, technologies, meant that the opposition they faced from some practitioners is unsurprising. Although, like much of the wider context, this contention is not discernible in the published medical literature, Russell remembered that the opprobrium which had been directed at Wickham in the early days of the PCNL re-emerged when he began operating on the gallbladder: "The antagonism that that man (Wickham) got from other people when he started removing stones through his little nephroscopes and things! You know, you’d have thought he was doing some crime, and we were in a similar position with gallstones". (Russell, personal interview, 22 January 2014)

Undeterred, Wickham, Kellett and Russell began their collaboration performing their first PCCL in 1986. They outlined their first successes with the PCCL in their 1988 *BMJ* article reporting that seven out of eight initial patients had undergone the operation successfully (with the remaining patient undergoing an open cholecystectomy after an adequate tract could not be made).^1^ Interest in the operation grew and in 1989 the BBC visited the team to film an episode of the BBC1 science documentary series *QED*. Entitled ‘Keyhole Surgery’, part of this episode showed Wickham and Kellett performing the PCCL and stressed the newness of this procedure.^24^ It was not only the public who were excited by the techniques shown; by this point there were other surgeons who were taking up the PCCL. As Russell recalled, "Certainly people came up and said to us, ‘Oh I’ve been trying that, it does work rather well". (Russell, group interview, 27 March 2015) Indeed, there are a few articles published around this time that reported successes with the PCCL, particularly for elderly or high-risk patients.^25–27^ The main concern for all the clinicians performing the PCCL though was the possibility of stones recurring in the gallbladder. For Kellett, Wickham and Russell this was a key concern. Two years after their 1988 *BMJ* article, they, along with other colleagues at the Institute for Urology, published again discussing their first 60 PCCL patients. Here, they noted that limited data on stone recurrence was available due to the newness of the procedure, although surmised that the recurrence rate would likely be similar to any other procedure where the gallbladder is left in situ. The article nonetheless ended on the hopeful note "We have shown the practicality of a less invasive method of removing biliary calculi that can complement both extracorporeal shock wave lithotripsy and cholecystectomy. Properly controlled trials of drugs that prevent recurrence of gall stones must now be carried out".^3^

This success, however, was to be short-lived and although the team continued developing the PCCL for around two more years, it would turn out not to be drug therapies or ESWL that would prove to be the future of gallstone surgery. As Kellett explained, ‘after that laparoscopic surgery had come in and people were taking out gallbladders through little holes … that was why the "pickle" (as the Americans called the PCCL) went out of date completely within months'. (Kellett, personal interview, 8 December 2014) The ‘pickle’— so called because of a phonetic pronunciation of the acronym PCCL rather than as a derogatory comment on the operation—indeed fell out of favour due to the emergence of the laparoscopic cholecystectomy, the minimally invasive removal of the gallbladder, one of the major success stories of minimally invasive surgery. It became clear very rapidly that the benefits of removing the entire gallbladder (especially in avoiding subsequent recurrence of stones) outweighed the drawbacks of postcholecystectomy syndrome.

Out of our initial interviews on the emergence of keyhole surgery, an untold story about medical innovation had emerged. PCCL was only mentioned occasionally by our interviewees, largely as a tangent to the much more successful PCNL. However, the story of the emergence, initial success and swift decline of the PCCL provides valuable insights into the development of minimally invasive surgery at the time. Inspired by the goal of minimising the trauma caused by open surgery, a group of clinicians worked together to adapt a successful procedure to a new area of the body. This entailed close work between surgical specialisms, radiology and the cultivation of relationships with industry. The importance of these interpersonal relationships is not obvious from the medical literature on the PCCL and only emerged through oral history interviews with the participants. However, surgery is a largely practice-based discipline and it is difficult to access this particular dimension through interviews. Therefore, in order to further flesh out the story and to better understand the practical aspects of the PCCL, we turned to simulation-based re-enactment. In developing a re-enactment scenario devoted solely to the PCCL, we wanted to immerse our interviewees in the history of that specific operation, to stimulate their memories further and to untangle the so-far somewhat tangential story of the PCCL from the larger story of minimally invasive surgery.

## Practical matters: re-enacting the PCCL

There have been several recent calls for historians to pay attention to issues of practice and skill in the history of medicine.^28 29^ Thomas Schlich for instance has argued that the changing performance styles of surgery over the 19th century—from the speed and force required of the surgeon in preanaesthetic times to the meticulous practices of the aseptic hospital—have much to tell us about the evolving character of surgery.^30^ With the emergence of minimally invasive procedures, the late 20th century also saw a significant change in the performance style of surgery, an element of the history that can be difficult to capture through traditional historical sources. For Schlich and others working on the 19th century practices must be reconstructed from texts or images. However, our work has recourse to a very different source; a group of living participants willing to once again don their scrubs and ‘perform’ operations—albeit using simulation.

Academic history has an uneasy relationship with historical re-enactment, often framing it uncharitably as the purview of military history obsessives in fancy dress.^12^ However, a growing body of literature on ‘re-enactment’ in historical research recognises the wealth of practices covered by the term.^31^ These range from hobby groups, ‘living history’ museums, and academic research methods in experimental archaeology to the reconstruction of historic materials, objects or practices to test theories about ancient life.^32 33^ Historians of science have used re-enactment as a tool with which to investigate topics such as 17th century alchemy and 19th century experiments with heat.^34 35^ Our own approach of simulation-based re-enactment (SBRE) makes use of simulation expertise within current medical education.^12^ Previously, we have used SBRE to re-enact open cholecystectomy with surgical teams who worked together during the late 20th century, in order to shed light on a once common operation that is no longer regularly performed today.^12^ Using SBRE to recreate the PCCL revealed the technical aspects of the operation and the more ‘tacit’ elements of surgical performance such as the individual styles of clinicians, social dynamics and teamwork in the operating theatre. Furthermore, it served as a prompt for our interviewees to discuss the PCCL in greater depth than they had in their initial interviews.

**Figure 1:**
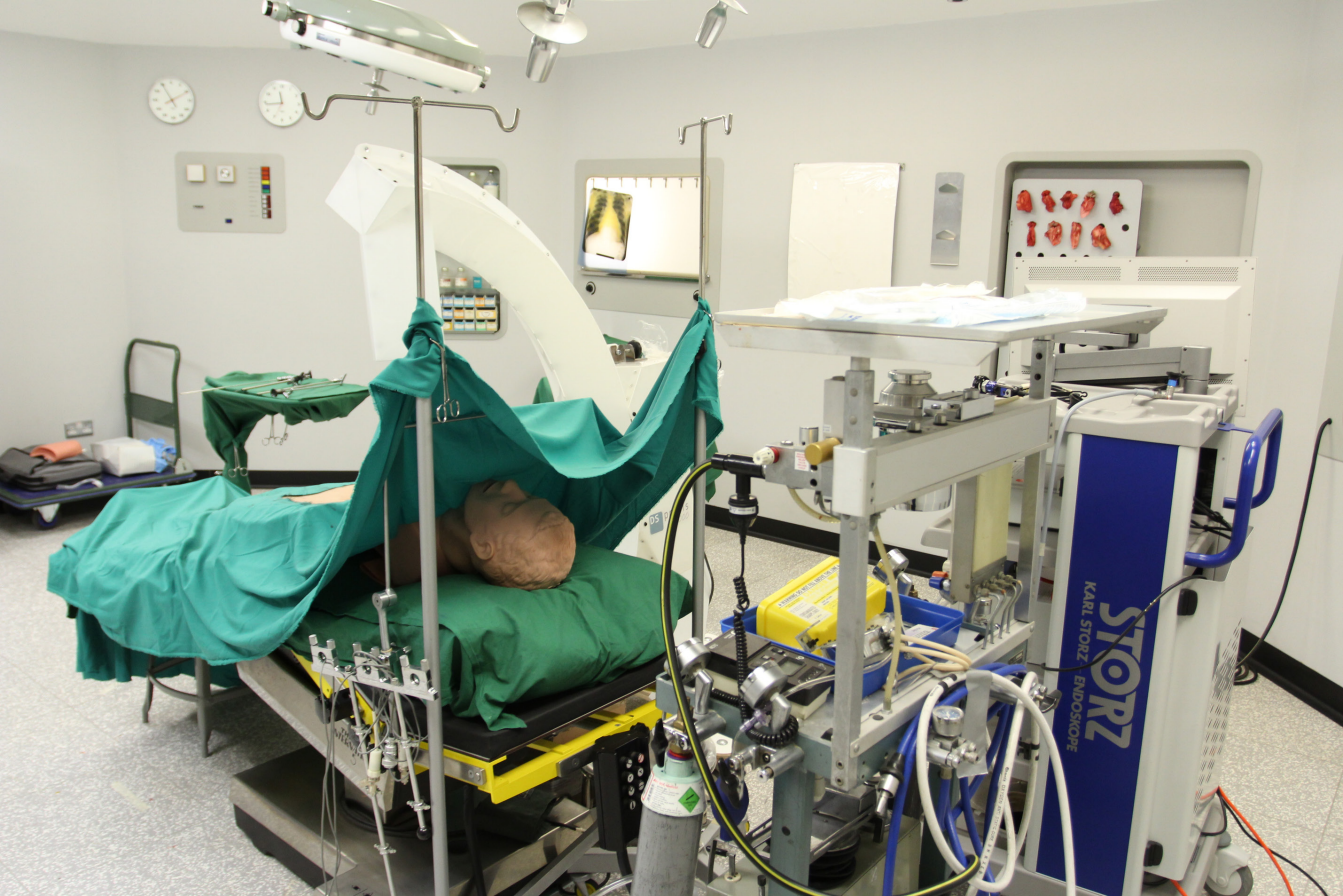
The Science Museum 1980s Operating Theatre set up ready for the percutaneous cholecystolithotomy. Photo credit: RLK.

For this study, we developed a PCCL re-enactment scenario. This took place in early 2015 at the Science Museum, London. At that time, the museum’s Lower Wellcome Gallery featured a life-sized replica of a fully equipped operating theatre, installed in the early 1980s. We augmented items from the museum’s collection with our own examples of 1980s surgical equipment, as well as a large number of original instruments from Wickham’s personal collection. The ‘patient’ was represented by a silicon body model, and the operative field was created from a cadaveric pig’s liver and gallbladder in which small stones representing ‘gallstones’ had been previously inserted by our team. In addition to Wickham, Kellett and Russell, the team included a contemporary scrub nurse and anaesthetist, while Greengrass was present as an observer. As in previous SBRE events, contextual realism was enhanced by using period-appropriate surgical scrubs, gowns and drapes, while recorded sound effects such as the whirr of a Manley ventilator provided additional verisimilitude (figure 1). Attention to these details is important in designing a useful re-enactment. The aim of the simulation-based re-enactment is to create an immersive experience for the participants. We did not want them to ‘act out’ the scenario but to come as close as possible to feeling like they were performing the surgery. In order to create as immersive an experience as possible, we needed the equipment and dressings to feel familiar to our clinicians.^12^ So, instead of the blue disposable surgical gowns used in modern-day operating theatres, we outfitted our clinical team in the heavy green cotton gowns used in the late 1980s. Despite some inauthentic elements, such as the presence of a two-person film crew in the ‘theatre’ and an invited audience of observers outside, the participants themselves seemed unaffected by these departures from routine. In the postsimulation discussions, Wickham and Russell were unconcerned.

Wickham: "I think it followed what we used to do pretty accurately, don’t you think?"

Russell: "Yes, I think so. It was similar to when we had visitors watching those operations. It reminded me of that exactly". (Group interview, 27 March 2015)

For the PCCL re-enactment, we invited Wickham, Kellett and Russell to perform the operation as closely as possible to their recollection of how they had done it years previously. The procedure began with Kellett creating and dilating a track into the gallbladder, followed by Wickham removing the stones. The laparoscope at that time was direct-viewing (not projected onto a screen as in later developments), and we were immediately faced with a very specific way of doing surgery. Bent over the eyepiece of his scope, Wickham’s whole focus was reduced to a tiny area of the patient’s body. As sociologists of medicine have noted, minimally invasive surgery totally transformed the surgeon’s relationship with the patient body as well as with their own senses.^36^ No longer able to see with the naked eye the structures on which they worked, surgeons had to relearn how to recognise anatomy through narrow telescopes and how to manipulate instruments and bodily tissues at a distance. This transformation in skill was immediately apparent during our simulation. However, it was not just the operating surgeon’s performance that the re-enactment revealed, other elements such as the transformation in the way operating teams worked also became evident.

From the late 19th century, surgery had remained largely unchanged in terms of its fundamental practice. Open operations with large incisions were the norm, rituals of sterility had been accepted in the form of handwashing, gloves, masks and gowns, and the hierarchy of the operating theatre—with the surgeon very much in charge—had changed very little. The 1980s though saw major transformations in these areas. Many once ‘bread-and-butter’ operations, such as cholecystectomy, were increasingly being replaced by drug treatments, radiological procedures and minimally invasive interventions.^37^ In the case of the cholecystectomy, we can see the increasing authority of the interventional radiologist. Radiology before the 1970s was largely devoted to the making and interpreting of diagnostic images; however, in the 1960s radiologists began experimenting with using their traditionally diagnostic technologies in treatment.^38^ We can see Michael Kellett’s work with John Wickham in developing the PCNL as one such example of this; what was once a radiological intervention to drain obstructed kidney’s developed into a way of carrying out a surgical procedure which, in turn, inspired them to trial the PCCL.

On the other hand, Russell’s role was supportive rather than active during the re-enactment, again highlighting the new forms of teamwork that were hinted at in our earlier interviews. Even though the gallbladder was technically Russell’s area of expertise, in the early days of the PCCL he would have learnt this new approach to the organ from Kellett and Wickham who had originally developed the technique on the kidney. Russell mentioned in an earlier interview that this sort of collaboration was not always popular, with some surgeons wanting to retain control over what they perceived as *their* areas. Russell though was dismissive of this attitude, explaining that if another surgeon, regardless of specialism, had developed a better way of operating then "you’re a fool not to follow. And so that was the obvious thing to do, and I would go along there and I’d see the patient and… I watched John doing it and I then got used to doing it, and that sort of thing". (Chris Russell, personal interview, 22 January 2014) In a period of increased specialisation, the sort of work that Wickham and Kellett proposed needed a group of practitioners who were happy to cross the boundaries of their specialisms, in this case a urologist, a radiologist and an upper gastrointestinal surgeon. From the re-enactment it emerged that Russell’s primary role was to provide security, in case the team needed to revert to open surgery because of unanticipated complications. In fact this was never necessary, but the presence of Russell provided confidence to the team as they explored their innovative approach.

As well as changes in style and teamwork, the re-enactment also highlighted environmental changes that we had not previously considered. For example, none of our surgical team wore masks during the re-enactment. Greengrass recalled that "[o]ne of the things that I learnt the first time I went to spend some time in theatre with John was that he didn’t treat it like an operating theatre, it was a procedure room. So he didn’t wear a mask". (Greengrass, group interview, 27 March 2015) Here, the re-enactment added further depth to our historical picture of the PCCL. Aside from the new physical skills demanded by such minimally invasive procedures, we also captured a glimpse into evolving ideas about sterility and the character of the operating space. It was the new collaboration between radiologists and surgeons that transformed the character of the traditional space of treatment. The PCCL necessitated a close working relationship between radiologist and surgeon with the radiologist becoming the practitioner who granted access to the interior of the body, traditionally the purview of the surgeon and scalpel. Therefore, the PCCL often took place in a radiological procedure room rather than a traditional operating theatre, and there was consequently little need for the surgical mask.

As well as allowing us to see these cultural and environmental changes, the re-enactment also prompted our participants memories of the PCCL, often to their surprise. For example, Greengrass said: "[f]or me that day was a portal, and it made me think about things I hadn’t thought about for a long time. But it triggered memories and insight that I’d long forgotten". (Greengrass, group interview, 27 March 2015) Russell agreed that the re-enactment had prompted reminiscences that the interviews had not. "Yes, I thought that it was ideal really. It’s the only way I would remember because that’s how my memory works, … it comes when you’re in the environment". (Russell, group interview, 27 March 2015) Oral history scholars have sometimes made use of objects or pictures in their interviews in order to stimulate the memories of their interviewees.^39^ Equally, the space in which oral history interviews are conducted is considered to have a significant impact on memory; if an interviewee is in a comfortable familiar space such as their home their recollections about their lives come more easily.^40^ Our re-enactment used and extended these practices, placing our participants in an environment that they would once have been intimately familiar with, surrounded by objects that they had once used on an everyday basis. Allowing for the busy schedules of our participants we arranged a group interview with Kellett, Wickham, Russell and Greengrass 6 weeks following the re-enactment to discuss the final part of the story—the decline of the PCCL, in more detail.

As some of the participants had noted in their earlier individual interviews, what swiftly put an end to the PCCL was the development of the laparoscopic cholecystectomy. This was the operation that marked the first major uptake of minimally invasive surgery by general surgeons. First performed in Germany in 1985 by surgeon Erich Mühe (1938–2005), the procedure was a technical success. However, Mühe faced indifference and resistance when he first presented his work and the laparoscopic cholecystectomy was largely ignored until the late 1980s.^8 41^ Many of Mühe’s detractors were concerned about the safety of this new laparoscopic surgery; however, there were other explanations for this initial apathy. One was the early successes of less invasive methods of treating gallstones, especially ESWL and chemical dissolution therapies. There was a general feeling that gallstone management was likely to become the work of internal medicine rather than surgery and that removing the gallbladder entirely would swiftly fall out of fashion.^42^ This was one of the same feelings that had motivated Wickham, Kellett and Russell; the possibility of a less invasive method than open surgery to remove a whole organ. By 1989, around the time Wickham, Kellett and Russell were finding success with the PCCL, the surgical world was just beginning to take the laparoscopic alternative to cholecystectomy seriously after further development of the operation by a team of French surgeons.^43^ Reports of these procedures finding success in France quickly began to reach the clinicians in the UK. Greengrass remembered hearing about it through industry links; he noted that "I was getting feedback about that through a little committee that I sat on with Olympus". (Greengrass, group interview, 27 March 2015) Likewise, word reached the clinicians and Russell and Kellett travelled to France to see the laparoscopic cholecystectomy performed, Russell recalled that this experience "just showed me that this was absolutely feasible, possible and a sensible way to go ahead". (Russell, group interview, 27 March 2015) With the removal of the gallbladder via the laparoscopic cholecystectomy clinicians also removed the possibility of stone recurrence, the main potential drawback of the PCCL.

In 1991, Russell coauthored an article reviewing all the currently available treatments for gallstones, including an extended section on the PCCL and a shorter section on the still-new laparoscopic cholecystectomy. In spite of the lack of formal reports, the initial successes were obvious, and Russell’s article concluded "[w]e believe that it is time to modify surgical technique to maintain cholecystectomy as the standard treatment for gallstones in the 1990s".^44^ His last journal publication on the PCCL was a 1994 *Gut* article which revisited the original 113 patients treated by Russell *et al* to evaluate the rates of gallstone recurrence in the years following their treatment. The article concluded that ‘[a] small group of patients continue to require alternative, non or minimally invasive methods, including oral dissolution therapy, extracorporeal shockwave lithotripsy or percutaneous cholecystolithotomy for the management of their gallstones’.^5^ The hope of clinicians in the 1980s had been that gallstone treatment would become less invasive than an open operation and medical professionals tried numerous different approaches. For internists this was dissolution therapy, whereas for some it was shockwave lithotripsy. For Wickham and Kellett having met with success in the kidneys with the PCNL, it logically became the percutaneous procedure. For others, the cholecystectomy was the only guarantee of preventing recurrence, so the issue was to reduce the trauma of that operation itself, by performing it laparoscopically. Of the two competing minimally invasive procedures, the laparoscopic cholecystectomy won out. There are multiple inter-related reasons for this; historians have noted the excitement generated among patients at the possibility of an operation that drastically reduced the length of stay in hospitals and pain associated with open surgery.^45^ Likewise, there was great excitement among surgeons about the laparoscopic procedure with general surgeons eager to begin performing the operation.^46^ In the medical literature, the key factor in stimulating this excitement was the reduced risk of gallstone reoccurrence. In 1995, Russell coauthored a chapter on ‘Percutaneous Management of Gallbladder Stones’ in a textbook of laparoscopic surgery, which noted that the stone recurrence rate was still ‘uncertain’, and stated that ‘the case for percutaneous management of gallbladder stones is weak in this age in which laparoscopic cholecystectomy is currently pre-eminent.^47^ Indeed, concerns about the stone recurrence rate would prove well-founded. A 2007 article reporting on a 10-year follow-up of 439 PCCL patients in Beijing recorded a stone recurrence rate of 41.46% and concluded with the lukewarm suggestion that PCCL be ‘considered carefully’ and combined with dissolution therapy to prevent recurrence.^48^ The almost immediate excitement over the laparoscopic cholecystectomy marked a quiet end to the PCCL for Wickham, Kellett, Russell and Greengrass. All moved on to different areas of experimentation and practice, encountering both successes and frustrations in their continuing development of minimally invasive surgery.^2^

## Conclusion

Innovations such as minimally invasive surgery emerged from complicated processes of trial and error. Not every procedure tried became a success and for every operation that became successful there were numerous procedures, like the PCCL, which encountered a dead end. Studying these ostensible failures is necessary in order to fully understand the processes of innovation. The PCCL is just one of these stories; as Greengrass noted following the re-enactment "[a]ctually I ended up slightly depressed because of all the things we tried, a lot of them at my instigation, which failed. I came out of that thinking; I’ve done an awful lot of stuff that hasn’t worked!" (Greengrass, group interview, 27 March 2015) It is a challenging task for the historian to uncover such ‘stuff that has not worked’ as failure is seldom written up in journals or textbooks. However it is far from impossible. Oral history provides an excellent tool with which to explore the motivations for trialling new operations and their subsequent development. Re-enactment offers a means of exploring the more tacit elements of the history, such as surgical performance, teamwork and environment that cannot be fully captured or made explicit through oral testimony. Re-enactment is also a powerful memory trigger for participants. Out of all these approaches emerges a cohesive story of the PCCL that would have been impossible to capture using textual sources alone. Russell, himself a former editor of the *British Journal of Surgery (BJS)*, remarked at the end of our final interview that "most of this would be edited out of the *BJS*, most of the information, because you were there to report science and not this nonsense! But actually science is made up of these small parts and this is probably where the stiff editor’s pen is less than ideal". (Russell, group interview, 27 March 2015) The history of the PCCL demonstrates that in uncovering the things that fell victim to the editor’s pen we can access a richer history of innovation, revealing the processes, practices and people at its heart.
